# The Phylacogen Treatment of Rheumatism*Read before Gross Medical Club, February 18, 1916.

**Published:** 1916-09

**Authors:** H. C. Lapp

**Affiliations:** North Tonawanda, N. Y.


### The Phylacogen Treatment of Rheumatism*

H. C. LAPP, M. D., North Tonawanda, N. Y.

In selecting this subject for our consideration this afternoon it is more for the purpose of bringing out a discussion concerning the merits or demerits of this new treatment than in attempting to offer anything new about the disease itself. For many years both among the laity and the profession it was a well accepted theory that rheumatism was caused by an excess of uric acid in the blood. While this condition might exist, it is not the cause but a result of the disease. In this day of scientific investigation it has come to be the accepted theory that rheumatism is caused by a specific organism. We may therefore define rheumatism as an infectious disease. There always has been a tendency among the profession to diagnose as rheumatism almost any pain in the joints or muscles, whether it be a neuritis, a gonorrheal arthritis, tubercular arthritis or a simple neuralgia. We are altogether too prone to tell our patients that they have a rheumatism when they come to us with a pain that is somewhat obscure and we are not sure as to the cause. In Forcheimer's Therapeusis rheumatism is defined as an acute febrile disease caused by the infection by specific organisms and characterized by an exudative, non-suppurative inflammation of the joints with a peculiar tendency to secondary involvement of the pericardium, endocardium and the heart muscle.

Bacteriologists have attempted to isolate and identify the specific micro-organism of rheumatism. Poynton and Payne claim to have isolated the germ and named it streptococcus rheumaticus. These claims have been confirmed by many other research workers, although they have not been accepted by some. Certain ones claim to have been able to produce arthritis and mitral disease in rabbits by the injection of the micro-organisms. It is believed that the tonsils and pharynx are very often the inroads of this infection. Langmead, from an investigation of one hundred and thirty-three cases, found that forty-three per cent. showed evidence of infection of the tonsils or pharyngeal necrosis.

It is on the theory that this disease is one of infection that the Phylacogen treatment is advocated. This treatment was originated in 1910 by Dr. A. F. Schafer, of Bakersfield, Cal. The principle upon which the use of Phylacogen is founded is the theory of multiple infection and that the growth of infection micro-organisms can be arrested and their effects

---

*Read before Gross Medical Club, February 18, 1916.

neutralized by products derived from their growth in artificial culture media. Rheumatism Phylacogen is composed of one-half Mixed Infection Phylacogen, which is prepared from a large number of species of the well known pathogenic bacteria, such as the several staphylococci, streptococci pyocyaneus, diplococcus pneumoniae, bacillus pyogenus, bacillus typhosis, bacillus coli communis, etc. These various organisms are present in the material before filtration in approximately the same proportions. These cultures are incubated at thirty-seven degrees centigrade for seventy-two hours and the bacteria killed, after which a preservative consisting of five-tenths of one per cent. of phenol is added to the fluid, which is then filtered through porcelain. To this Mixed Infection Phylacogen is added an aqual amount of the filtrate of the streptococcus rheumaticus and the resulting preparation is called Rheumatism Phylacogen.

Phylacogen is administered in two ways, either subcutaneously by hypodermic injection or intravenously by direct injection into a vein. I have never tried the intravenous method. My experience in the treatment of these cases has been seven, of which the most interesting to me was a personal one. I wish to report these cases with the method of treatment and the result. In each case the Phylacogen was given after seemingly everything else had failed.

If you will pardon reference to my own case, wish to state that I was taken sick in the early part of April, 1913, and called in some of my medical colleagues and was treated by them for seven weeks following the usual methods. I was given all the Rheumatics known and seemed to receive no relief. We finally decided to try the Phylacogen treatment. On the first day I was given two c.c. of the Phylacogen subcutaneously and repeating the injection on each succeeding day, increasing one c.c. each day until I was taking ten c.c. at each dose. This was continued until I had received fifty c.c. There was no noticeable reaction until the dose was increased to 5 c.c. or more. On the fourth day after beginning the treatment I began to have less pain, and swelling and redness began to disappear. In a week I was up and continued to improve until I was entirely well and have continued so ever since, a period of about two and one-half years.

I would like here to describe the reaction which takes place following a large dose of Phylacogen. In about three or four hours after the injection there is quite a distinct chill lasting about fifteen minutes, which is followed by a sharp rise of temperature lasting for several hours. This is followed by some depression and loss of appetite. Favorable results may be obtained by giving small doses repeated daily and gradually increasing according to the tolerance of the individual. We do not get the depression and severe reactions following this plan of treatment. In all the cases except my own the small series of doses was used.

Case 1.—The first case that I treated was a woman, age 24, married, gave a history of having suffered from rheumatism for five years. Had been treated by several different physicians with no benefit. I started the treatment on June 11, 1914, at which time she was suffering with pain in the ankles and feet with swelling and redness of the joints. I started by giving two c.c. of Rheumatism Phylacogen subcutaneously, giving her an injection each day, increasing the dose until I gave her five c.c. at one dose. I did not go above five c.c. in this case. In all she had fifty c.c. of the Phylacogen. On the fifth day there was an improvement both in the pain and in the swelling, and patient was able in a week to walk and move the joints without pain. There has been no return in this case after a year and a half.

Case 2.—Man, age forty-five, gave a history of repeated attacks of inflammatory rheumatism for the past fifteen years, had a very bad valvular disease of the heart. Had not been able to do any work for ten years. History of slight stroke of paralysis five years before. Was taken with an acute attack of inflammatory rheumatism and after a few days of other treatment without benefit it was decided to try the Phylacogen. I gave two c.c. subcutaneously each day for several days and the heart seemed to be more steady. The pain and swelling was not so acute and patient was apparently making a good recovery from his acute attack when about one week after I had finished with the Phylacogen treatment the man suffered another stroke of paralysis which involved his whole right side. This was followed by an ascites and at the end of a week the man died. The Phylacogen had nothing to do with the cause of the cerebral hemorrhage as the treatment had been discontinued for a week.

Case 3.—Woman about 38 years of age had been suffering from chronic articular rheumatism with beginning arthritis deformans. Patient had been treated for several months by another physician and was turned over to me for treatment with the Phylacogen. I began treatment in this case on September 30th, 1914, and continued for ten days, giving her repeated small doses of Phylacogen. When I began the treatment patient was in bed and had a great deal of pain and deformity of the hands and fingers. At the end of ten days she was able to be up about the house, at which time I was discharged. This case was not cured and she stopped the treatment just when it was needed most. At present the patient is taking osteopathic treatments for rheumatism. I

believe that this case would have been greatly benefited if she had continued the Phylacogen treatments.

Case 4.—Woman, age 47, has been suffering from rheumatism for over two years, has been treated by several physicians with no relief, and her condition appearing to grow worse each day. For four months she was treated by an osteopath. I was called to see the case November 21, 1915. Her condition was pitiable, she was unable to walk or feed herself, all joints of the legs and feet and also right wrist and elbow badly swollen and inflamed. Was unable to sleep without opiates. I started this case by giving one c.c. of Phylacogen subcutaneously and increasing to two c.c. on the third day. I continued the treatment for about five weeks, giving an injection every day for about three weeks and every other day thereafter. For three or four weeks there was a distinct improvement both in the patient's general condition and in the rheumatism. Her appetite improved and after about ten days she was able to be up in her chair during the entire day. The cramps in her limbs left and she was able to sleep well. Beyond this point there did not seem to be an improvement as the function of the joints was not restored owing to considerable permanent damage to the parts involved. The woman at the present time is able to be up all day, eats and sleeps well, has very little pain, but is unable to walk and has very little use of her hands. I am unable to say whether more Phylacogen would have improved this condition as the patient became discouraged and decided to discontinue the treatment.

I am at the present time treating two cases of acute inflammatory rheumatism with Phylacogen. In both cases I treated patients for two weeks with salicylates and other medicinal treatment. But there was a steady increase of pain and involvement of joints of each case. In one of these I had an involvement of the heart with great weakness. I stopped all medicines except heart stimulants and started giving one c.c. of Phylacogen each day, increasing on the third day to 2 c.c. I have been giving the Phylacogen for a week now, the patient is sitting up about two hours each day, eating well, and is apparently making a splendid recovery. The other case is out of bed and dressed every day now, free from pain and is eating well. I did not give three c.c. in either one of these cases. In neither of them has the patient over thirty c.c. of the Phylacogen.

While I do not believe that we will get results in all cases, and while those I have treated have been few in number, the results of those treated have been such that I believe that in a majority of cases of true rheumatism we have in Phylacogen a remedy that if properly administered will serve us well.

270 Payne Avenue.

---

**Diabetes.** (Condensed from review by H. C. King, Cleveland Med. Jour., April), F. M. Allen, Am. Jour. Med. Sci., Oct., 1915, emphasizes the dangers of fat in producing coma and holds that acid bodies cannot be formed from carbohydrates. (Note: Of course, this does not apply to action by bacteria as in gastroenteric or even buccal fermentation nor is strictly true even for catabolic changes for carbon dioxid is essentially an acid and there is some evidence that acidosis in the clinical sense depends largely on the ultimate balance of reaction in the system, in which carbon dioxid is the largest factor). Glycosuria in itself is not so important as acidosis.

Edgar Stillman, ibid. April, 1916, holds that urinary tests for acidosis are often misleading because they measure only the excretion and not the retention. He approves Van Slyke's simplified test (not yet published) for determining the bound carbon dioxid in blood plasma. (Note: However, the direct acidity of the urine, if excessive and maintained for several days is a pretty good index of the general balance of the system).

Elliott P. Joslyn, Am. Jour. Med. Sci., Mch. 1916, holds that large doses of sodium bicarbonate are frequently dangerous in threatened coma. (Note: This is a point which may be questioned. If true it is exceedingly important). He thinks that fasting should be preceded by absolute exclusion of fat from the diet, then the omission of proteid, then successively cut the carbohydrate in halves till 10 grams are reached. (Note: A fasting patient is really on a diet of proteid and fat, probably mainly fat, after the small store of glycogen is exhausted). He tabulates the essential cause of death in 420 diabetics: 147 died without and 273 with coma. Of the 147, cancer caused the death of 17, cardio-vascular-renal disease of 62, including 14 cases of cerebral haemorrhage. 16 died of pulmonary tuberculosis. Infections caused death in 36. Pneumonia caused 15 deaths, amygdalitis 11, gangrene 9. "Numerically infections take front rank by precipitating coma for over 78% of the deaths." (Note: As we understand the somewhat contradictory explanation, the 143 cases stated to have died without coma, included many that died in coma due to an infection. This brings up the question whether "coma" can be exactly defined).

